# Nanoporous PLA/(Chitosan Nanoparticle) Composite Fibrous Membranes with Excellent Air Filtration and Antibacterial Performance

**DOI:** 10.3390/polym10101085

**Published:** 2018-09-30

**Authors:** Hui Li, Zhe Wang, Haiyan Zhang, Zhijuan Pan

**Affiliations:** 1 College of Textile and Clothing Engineering, Soochow University, Suzhou 215021, China; lh1433950564@163.com (H.L.); wang_zhe@ntu.edu.sg (Z.W.); 18862115625@163.com (H.Z.); 2 National Engineering Laboratory for Modern Silk, Soochow University, Suzhou 215123, China

**Keywords:** electrospinning, PLA, chitosan NPs, filtration, antibacterial

## Abstract

Particulate matter (PM) pollution, which usually carries viruses and bacteria, has drawn considerable attention as a major threat to public health. In this present study, an environment-friendly antibacterial Poly(lactic acid)(PLA)/chitosan composite air filter was fabricated using the one-step electrospinning technique. The composite PLA/chitosan fibres show a highly porous structure, in which chitosan nanoparticles (NPs) were found to be uniformly distributed throughout the entire fibre. The morphologies, through-pore size and distribution, air filtration and anti-microbial properties of these filter media were studied. The results showed that it was not the chitosan content but instead the concentration of the spinning solutions that had the greatest effect on the morphologies of the porous fibres. The relative humidity influenced the nanometre-scale pores on the surface of PLA/chitosan fibres. The PLA/chitosan fibrous membranes with a chitosan to PLA mass ratio of 2.5:8 exhibited a high filtration efficiency of 98.99% and a relatively low pressure drop (147.60 Pa) when the air flow rate was 14 cm/s, while these also had high antibacterial activity of 99.4% and 99.5% against *Escherichia coli* and *Staphylococcus aureus*, respectively. It took 33 min for the PM_2.5_ concentration to decrease to 0 μg/m^3^ from 999 μg/m^3^ using the PLA/chitosan fibrous membranes, which demonstrates obviously effective air purification performance.

## 1. Introduction

In recent decades, the levels of particulate matter (PM) in the air, which are well known to affect the quality of human life and even to pose a serious threat to the health of the public [1,2,3,4,5,6,7] have been obviously rising due to rapid urbanization and industrialization. In most situations, the components of indoor air are complex, including solid particles, liquid droplets, pet hairs, etc., and can pose great harm. Previous studies have shown that PM_2.5_, which are particles with diameters that are less than 2.5 μm, are particularly harmful because they can penetrate human bronchi, lungs and even extrapulmonary organs [1,8,9]. In addition, PM_2.5_ always carries and spreads bacteria and viruses, causing serious harm [9]. Therefore, filter media with excellent filtration and antibacterial performance are in high demand.

Recently, various types of polymers have been fabricated as electrospun fibrous membranes to be used as air filtration media. Nanofibre membranes with optimal characteristics, such as a high specific surface area and porous microstructure with interconnected pores, can efficiently capture the fine particles [8,10,11,12]. Electrospun mats have also been widely researched in the field of biomedical materials, such as tissue engineering scaffolds, drug delivery, etc. [13,14,15]. Electrospinning is an efficient and versatile continuous process for the preparation of uniform nanofibres and the technique is becoming increasingly mature after years of research. In recent years, various types of polymers have been fabricated as electrospun fibrous membranes to be used as air filtration media, such as PVA [16], Nylon 6 [17] and PAN [8,12,18]. Most of them are petroleum-based polymers, the application of which has been found to coincide with serious environmental pollution. Additionally, fossil resources are gradually being depleted [19].

Poly(lactic acid) (PLA, [CH(CH_3_)COO]_n_) is a linear aliphatic polyester, which can be prepared from renewable bioresources through fermentation and polymerization [15]. In recent years, PLA has drawn research attention worldwide due to its good biocompatibility and biodegradability [20,21]. In addition, PLA can be dissolved in common solvents and turned into nanofibrous membranes with good mechanical properties through electrospinning. Bognitiski et al. were the first to fabricate nanostructured, electrospun PLA fibres using a dichloromethane (DCM) solvent [22]. Zhe Wang et al. used PLA as a raw material in the manufacture of porous nanofibre membranes, which displayed excellent air filtration performance [23,24].

Chitosan has attracted great interest as a new functional biomaterial because of its advantageous properties, such as biodegradability, biocompatibility [25], nontoxicity and natural antimicrobial activities against different groups of microorganisms [26,27]. However, the electrospinning of chitosan is difficult due to its limited solubility in most organic solvents as well as its high viscosity and strong hydrogen bonds in acidic aqueous solutions [28,29], which has resulted in chitosan having limited applications as an electrospun fibre material. Therefore, chitosan and its derivatives should be blended with a polymer solution before electrospinning. Polymers that can be dissolved in acidic aqueous solutions are preferred, such as PEO and PVA [30,31].

Electrospun PLA/chitosan nanofibres are expected to form a new biomaterial that possesses a combination of properties, which cannot be exhibited by individual polymers. During recent decades, significant progress has been made in the preparation of PLA/chitosan nanofibres [32,33,34,35,36,37,38]. The results show that the differences in nature, structure and compatibility between PLA and chitosan lead to a poor dispersion of the two phases. Nguyue et al. [39] even used PEO as a compatibilizer in order to improve the interaction and dispersion between PLA and chitosan phases. Andri et al. [14] added ethanol to make PLA/chitosan solutions miscible without precipitation. However, these measures did not appear to work well.

In this study, chitosan NPs were added to PLA solutions and were dispersed homogeneously without affecting the solution viscosity. Fibres made from the suspension by electrospinning had nanopores on their surfaces and the chitosan was distributed on the surfaces of the fibres in the form of particles. The membranes were characterized with a field emission scanning electron microscope (FE-SEM), energy-dispersive X-ray spectrometer (EDX), a transmission electron microscope (TEM), a through-pore size analyser and automated filtration testing unit. The antibacterial activity of the PLA/chitosan composite fibrous membranes was evaluated according to GB/T 20944.3-2008: Textiles–Evaluation for antibacterial activity—Part 3: Shake flask method.

## 2. Materials and Methods

### 2.1. Materials

PLA (Wm = 1.0 × 10^5^, Melt Index (190 °C, 2.16 kg) = 1–30 g/10 min) was obtained from Zhejiang Hai Zheng Biological Materials Co., Ltd., Taizhou, China. Chitosan NPs (degree of deacetylation = 95%, viscosity = 42 cps) with a diameter of 100–600 nm were supplied by ShanDong LaiZhou Highly Bio-Products Co., Ltd., Laizhou, China. Both *N,N*-dimethylacetamide (DMAC) and dichloromethane (DCM) were purchased from the National Medicine Group Chemical Reagents Co., Ltd., Suzhou, China. All of the chemicals were of analytical grade and were used without further purification.

### 2.2. Preparation of Solutions

A mixture of DCM/DMAC (with a mass ratio of 10:1) was used as the solvent for the PLA. Initially, the PLA polymer was dissolved by stirring for 12 h at room temperature. After that, the chitosan NPs were added to the as-prepared PLA solutions by stirring for 2 h, with these dispersed NPs having maintained their shape without being dissolved. The solutions were then sonicated for 90 min to obtain a well-mixed homogenized suspension.

As listed in Table 1, one set of samples (set A) was composed of PLA solutions of different concentrations (4 wt %, 5 wt %, 6 wt %, 7 wt % and 8 wt %), while the mass ratio of chitosan NPs to PLA was 1:4 (*w*/*w*). The other one (set B) was composed of PLA solutions with 8 wt % concentration containing different mass ratios of chitosan NPs (the chitosan NPs to PLA ratios were 0:8, 1:8, 1.5:8, 2:8 and 2.5:8 (*w*/*w*)).

### 2.3. Electrospinning

The prepared PLA/chitosan solution was loaded into a 5-mL syringe with a 21-gauge needle tip that was connected to a voltage supply (DWP503-1ACDF, Tianjin Dongwen High Voltage Co., Tianjin, China) and ejected at a controllable feed rate (1 mL/h), which was operated by a syringe pump (KDS-100, KD Scientific Inc., Holliston, MA, USA). A high voltage (18 kV) was applied to the needle tip. The composite PLA/chitosan fibres were deposited on the nonwoven fabrics wrapped around a grounded metallic roller rotating at 5.5 m/min, which was positioned 14 cm from the tip of the needle.

### 2.4. Characterisation

#### 2.4.1. Solution Viscosity

The viscosity of the PLA and PLA/chitosan solutions was measured with a rheometer (AR 2000, TA Instruments, New Castle, DE, USA) at 20 °C. The diameter of the cone plate was 40 mm and the shear rate was 0–1001 s^−1^.

#### 2.4.2. Fibre Morphology

The FE-SEM (S-4800, Hitachi Ltd., Tokyo, Japan) images of the electrospun fibres were obtained under an accelerating voltage of 3 kV. The dispersion of chitosan NPs in the fibres was examined using a TEM (HT7700, 120 kV, Hitachi Ltd., Tokyo, Japan). The average fibre diameter and average diameter of the pores on the surface of the micro-scale fibres were measured by using image processing software (Image-Pro Plus 5.0) and were tested 100 times and 300 times, respectively.

#### 2.4.3. Elemental Detection

EDX analysis combined with SEM (TM3030, Hitachi Ltd., Tokyo, Japan) was used to detect the element concentrations and distribution on the electrospun fibrous membranes at randomly selecting areas, before X-ray net counts were obtained.

#### 2.4.4. Through-Pore Size and Distribution

The through-pore size and through-pore size distribution of the fibrous membranes were measured by using a manually operated through-pore size analyser (Porometer 3G, Quantachrome Instruments Co., Boynton Beach, FL, USA). The fibrous membranes were cut to a diameter of 2.5 cm and thoroughly wetted with a liquid accessory kit (Number 01150-10035) to fill all of the pores with liquid (Porometer 3G—Porofil^®^ Wetting Solution). The wetted sample was subjected to increasing pressure from a gas source. As the pressure of the gas increased, the liquid in the largest pores was pushed out. Increasing the pressure further allowed the gas to flow through the smaller pores until all of the pores were emptied and thus, a ‘wet’ run was obtained. When the dried sample was then tested without liquid, a ‘dry’ run was obtained. Comparing the flows from the ‘wet’ run with those from the ‘dry’ run, the through-pore size and through-pore size distribution could be calculated.

#### 2.4.5. Filtration Performance

The aerosol filtration efficiency and airflow resistance of the composite PLA/chitosan fibrous membranes were measured with an automated filtration testing unit (TSI8130, TSI Inc., Shoreview, MN, USA). NaCl aerosol particles with a 260-nm mass median diameter and a 75-nm count median diameter were prepared with an aerosol particle generator. The neutralized NaCl aerosol particles were fed into a filter holder and passed through a filter with an effective area of 100 cm^2^. All NaCl aerosol tests were conducted at room temperature at a continuous face velocity of 14 cm/s.

Quality factors (QF) were generally used as the comprehensive parameter to evaluate the filtration performance of the filter. QF were defined as QF=−ln(1−η)/Δp, where η and Δp refer to the filtration efficiency and the pressure drop, respectively.

#### 2.4.6. Antibacterial Activity

The antibacterial activity of the composite PLA/chitosan fibrous membranes against *Escherichia coli* and *Staphylococcus aureus* was evaluated using a viable cell-counting method, which is described below, according to GB/T 20944.3-2008. Here, both *E. coli* and *S. aureus* bacterial species were first activated with a fresh nutrient agar medium, before being incubated overnight (18–24 h) at 37 °C. Subsequently, the typical colonies were placed in 20 mL of nutrient broth and were incubated at 37 °C with gentle shaking (130 rpm) for 18–20 h. After this, the bacterial solution was diluted to a concentration of approximately 4 × 10^5^ CFU/mL. Typically, the sterilized PLA/chitosan fibrous membranes with a weight of 0.75 g were exposed to the bacterial solution for 24 h at 24 °C under visible light. The bacterial solutions were then inoculated in Petri dishes containing the nutrient agar after ten-fold dilutions at 37 °C for 24 h. The average number of surviving bacteria was determined by counting the colonies on the three incubated agar plates for each sample. The antibacterial activity was evaluated based on

(1) $$\mathrm{Antibacterial}\;\mathrm{activity}\;=\frac{\left(N_{\mathrm{control}}-N_{\mathrm{sample}}\right)}{N_{\mathrm{control}}}\;$$

where $N_{\mathrm{control}}$ and $N_{\mathrm{sample}}$ are the quantities of the visual bacterial colonies on the standard cotton fabric and the tested PLA/chitosan composite fibrous membranes, respectively.

#### 2.4.7. Air Purification Performance

In order to evaluate the air purification performance, a filter element for a car air purifier (HS3122, Husum Technology Co. Ltd., Shanghai, China) was prepared using the membrane, with the area maintained at a consistent value with the filter included with the purifier. A polluted environment was created with cigarettes in a confined space with a volume of 0.125 m^3^, before the vehicle filter was placed in the polluted space. The PM_2.5_ concentration was tested by an air quality detector (MEF-550, Sensology Institute, Michigan, MI, USA). The time taken for the concentration of PM_2.5_ to decrease to 0 μg/m^3^ from 999 μg/m^3^ was recorded, which was an indication of the air purification performance of the fibrous membranes.

All the membranes used for through-pore size, filtration and air purification performance tests were prepared under the same conditions, including the spinning time (6 h) and the humidity.

## 3. Results and Discussion

### 3.1. Morphologies of PLA/Chitosan Porous Nanofibres

#### 3.1.1. Effect of Concentration on Morphology of PLA/Chitosan Fibres

**Figure 1:**
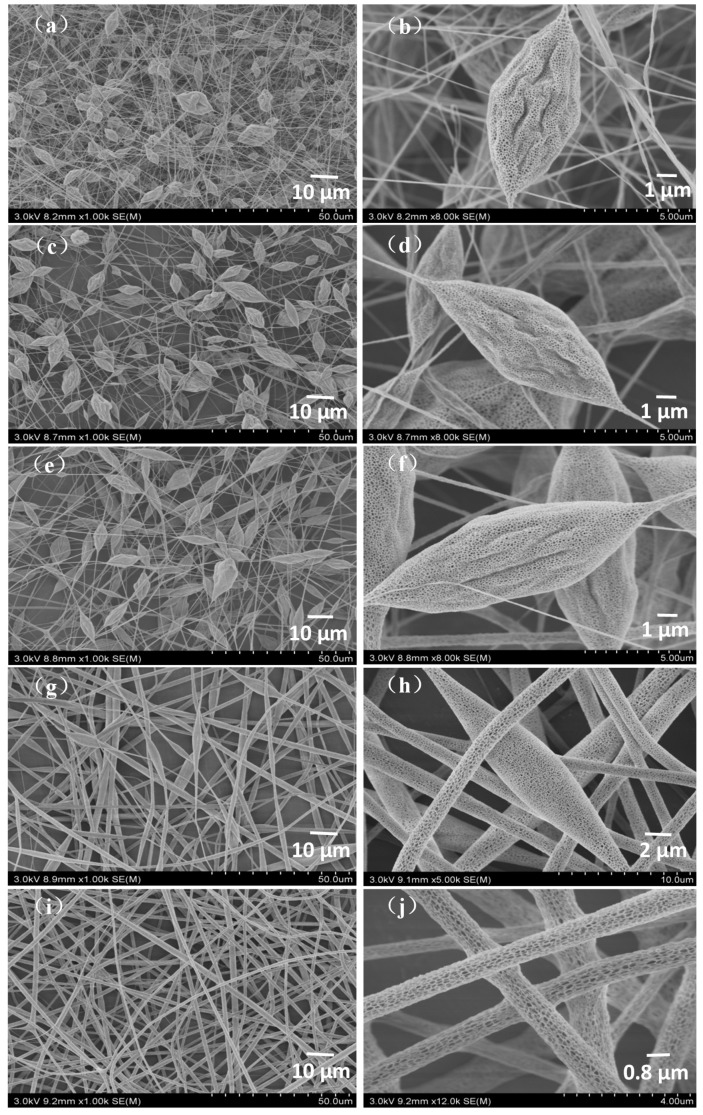
FE-SEM images of the PLA/chitosan fibrous membranes prepared at different concentrations: (**a**,**b**) 4 wt % PLA, 1 wt % CS; (**c**,**d**) 5 wt % PLA, 1.25 wt % CS; (**e**,**f**) 6 wt % PLA, 1.5 wt % CS; (**g**,**h**) 7 wt % PLA, 1.75 wt % CS; and (**i**,**j**) 8 wt % PLA, 2 wt % CS.

**Figure 2:**
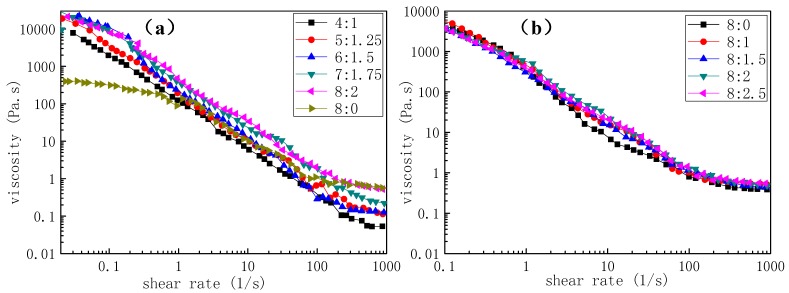
Viscosity of the solutions: (**a**) solutions with different concentrations; and (**b**) solutions of 8% PLA containing various ratios of chitosan.

Concentration has a significant influence on the morphology of fibres. The FE-SEM micrographs of PLA/chitosan nanofibres fabricated from the PLA/chitosan solutions (the weight ratio of chitosan to PLA was 1:4) under 45% humidity are presented in Figure 1. Bead-on-string fibres were obviously observed when the mass fractions of PLA in the mixtures were 4%, 5% and 6% (Figure 1a–f). The number of the beads gradually decreased and the wrinkles on the beads smoothened as the concentration increased. When the concentration of PLA in the solution reached 8 wt %, the fibres became even with no beads present (Figure 1i,j). The reason for this phenomenon was that the increased solution concentration led to changes in the properties of the solutions, especially viscosity. As shown in Figure 2a, the viscosity increased with the increase in the concentration of polymers, which was the main factor resulting in changes to the fibre morphology. As clearly displayed in Figure 1b,d,f,h,j, many mesopores and macropores (with a width ranging from 29 nm to 107 nm) were distributed on the surface of the beads and bead-less fibres, which resulted from the phase separation and breath figure induced by the rapid solvent evaporation and vapor penetration and condensation [40]. It is generally accepted that the pores on the surface of fibres affect the specific surface area and the nanoporous structure of the resultant fibrous membranes [24,41,42], which ultimately affect the air filtration efficiency and purification performance [43]. Additionally, no chitosan NPs were observed on the surface of the fibres in Figure 1 (×4k, 5k and 10k) and it was speculated that the chitosan NPs could be broken up into smaller particles under the high-voltage electric field during the electrospinning process, before being distributed on PLA fibres [15].

#### 3.1.2. Effect of Humidity on Morphology of PLA/Chitosan Fibres

**Figure 3:**
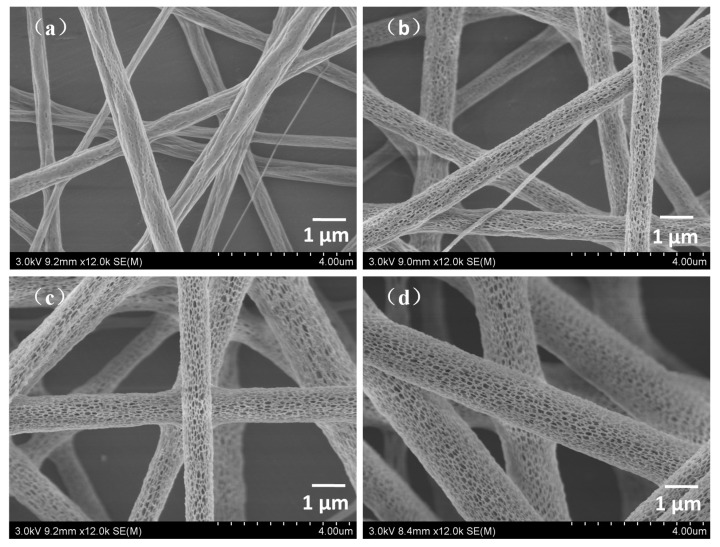
FE-SEM images of the PLA/chitosan fibrous membranes fabricated at different levels of humidity: (**a**) 15%, (**b**) 30%, (**c**) 45% and (**d**) 60%.

Water droplets are most likely to be the cause of pore formation on the fibre surface so humidity may significantly affect the nanopore size and number because much more vapor will distribute into the air when the relative humidity is high. To determine the effects of humidity on the nanopores and the properties of the fibrous membranes, fibres with different pore structures (Figure 3) were fabricated with a solution at a PLA/chitosan mass ratio of 8:2. In this experiment, we varied the humidity from 15% to 60% while the other parameters were kept constant. As seen in Figure 3a, the surface of the fibres prepared at a humidity of 15% was nearly smooth with an average fibre diameter of 0.91 μm (Table 2). When the humidity increased to 30%, many pores were discovered and the average diameter increased to 1.15 μm. Under 45% and 60% humidity, the nanopores became denser and deeper, while the diameters increased to 1.34 μm and 1.50 μm, respectively. As displayed in Table 2, the average surface nanopore width increased from 46.0 nm to 65.8 nm and the surface nanopore coverage ranged from 18.13% to 32.72% when the humidity was varied from 30% to 60%.

All the results above show that the humidity had a significant effect on the formation of nanopores on the fibre surface, which is consistent with the conclusions of previous reports [24]. It could be interpreted that a high relative humidity would result in much more vapor in the air and thus, a large number of water droplets form on the surface of the solution jets and penetrate the jet fluid [44,45,46].

#### 3.1.3. Effect of Mass Ratios of PLA and Chitosan on Morphology of Fibres

As reported previously [47], the type of additive may have an effect on the morphology of the polymer fibres. For instance, chitosan dissolved in the solution could increase the charge density of the polymer jet, resulting in a smaller diameter of the fibres [14]. In order to identify whether the chitosan NPs affected the morphology of the fibres or not, PLA/chitosan fibres with different chitosan (CS) contents (the ratios of chitosan NPs to PLA were 0:8, 1:8, 1.5:8, 2:8 and 2.5:8) were prepared and analysed by FE-SEM. As reported in Table 3, the diameter of the PLA/chitosan fibres was larger than that of pure PLA fibres. However, the morphologies of fibres with different levels of chitosan showed no obvious distinction, including in the surface nanopore width and coverage. This shows that the increase in the chitosan content had little effect on the viscosity (Figure 2b) of the solutions, as reflected by the fewer changes in morphology. Moreover, dispersing the chitosan NPs into the solution to fabricate the electrospun fibres made the process simple and easy to control.

### 3.2. Dispersion of Chitosan NPs and Element Contents

**Figure 4:**
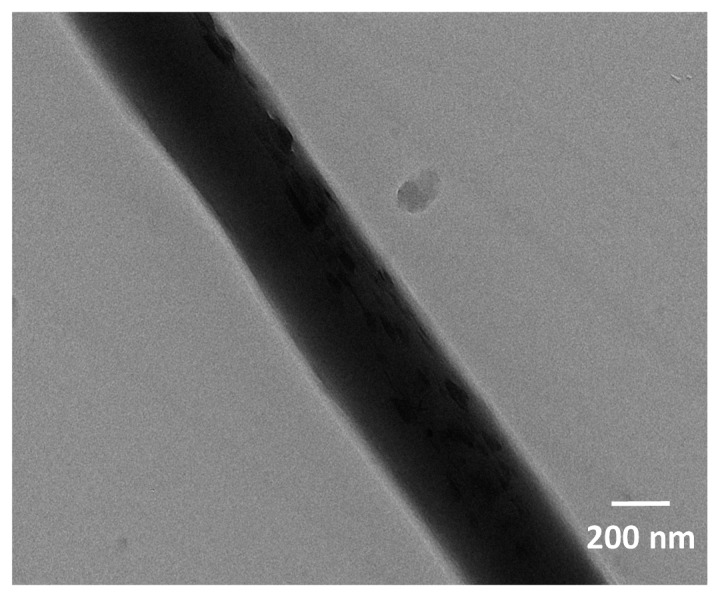
TEM image of PLA/chitosan fibres.

**Figure 5:**
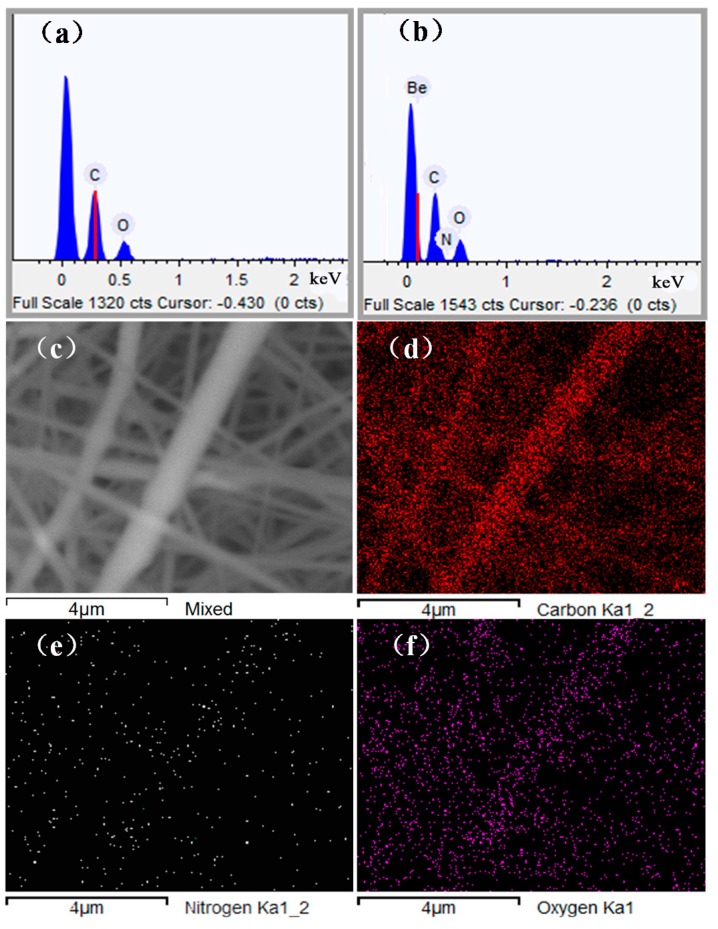
EDX element analysis: (**a**) PLA fibres and (**b**) PLA/chitosan fibres; (**c**) SEM image of PLA/chitosan membrane, EDX mapping of electrospun PLA/chitosan membrane: (**d**) C, (**e**) N and (**f**) O elements on the surface of the fibres.

The TEM image of the PLA/chitosan fibres is displayed in Figure 4. It can be observed that when the chitosan concentration was 20% in the fibre, the chitosan NPs were evenly inlaid on the surface of the fibre without agglomeration. It is important to note that the chitosan NPs were very small, with sizes of just tens of nanometres. This proved that the hypothesis in Section 3.1.1 is reasonable. SEM-EDX analysis was used to investigate the chemical composition and relative distribution of chitosan on the surface of PLA/chitosan fibrous membranes. Figure 5 shows that the pure PLA fibres contained carbon (C) and oxygen (O), but no nitrogen (N). When the chitosan was added to the membranes, N was also detected. The membranes contained 2.75 wt % of N as the content of chitosan was up to 20 wt % in PLA/chitosan fibres (Table 4), which also confirmed that the chitosan NPs were successfully loaded onto the PLA fibres. The EDX mapping (with the red colour corresponding to C, the white colour corresponding to N and the purple colour corresponding to O) in Figure 4d–f shows the distribution of N on the fibres, indicating that the chitosan particles were uniformly distributed along the PLA fibres.

### 3.3. Through-Pore Size and Distribution

**Figure 6:**
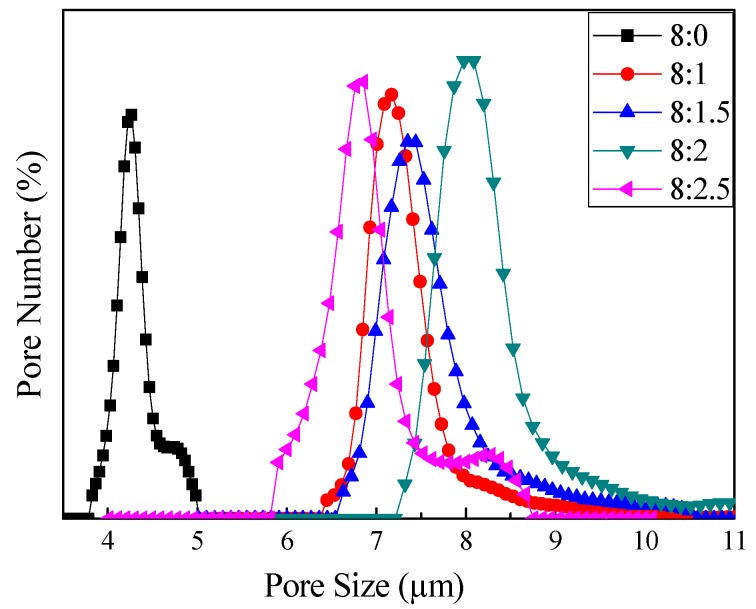
Through-pore size and through-pore size distribution.

The through-pore size and through-pore size distribution of the nanofibre membranes are very important parameters in determining the air filtration performance of the fibrous filter. The results shown in Table 3 clearly indicated that the addition of chitosan increased the mean flow through-pore size of the fibrous membranes from 4.5 μm to more than 7 μm, while the distribution of the through-pore size became slightly broader (Figure 6). However, the mean flow through-pore sizes between the membranes varied slightly with any further increase in the chitosan contents, which was the same as in the case of the fibre diameter.

### 3.4. Filtration Efficiency

The filtration efficiency and pressure drop of the fibrous membranes are the main properties that are important for air filtration. They were evaluated by filtering NaCl aerosol particles with a mass median diameter of 260 nm, which is shown in Table 5. The commercially available filter element FY3107 (NanoProtect filter Pro S2, FY3107, Royal Philips Electronics of the Netherlands, Netherlands) was tested as a control. It can be observed that the filtration efficiency of nanofibre membranes is significantly greater than that of the filter purchased (89.18%). This result could be because of the high specific surface area and the small through-pore size of nanofibre membranes. Compared to the pure PLA membrane (99.90%), the filtration efficiency of PLA/chitosan fibrous membranes was slightly lower at 98.10–98.99%, whereas the chitosan content had almost no effect on the filtration efficiency. However, the pressure drop of pure PLA membrane was 335.90 Pa and it decreased to 167.05 Pa when the mass ratio of chitosan to PLA was 1:8. These phenomena can be attributed to the variation of fibre diameter and through-pore size of the membranes (Table 3). As the fibre diameter and the through-pore size increased, the corresponding filtration efficiency and pressure drop decreased. According to the quality factor, when the mass ratio of chitosan to PLA was 2.5:8, the quality factor (up to 0.0312) was the highest, which indicated the best filtration performance.

### 3.5. Antibacterial Activity

**Figure 7:**
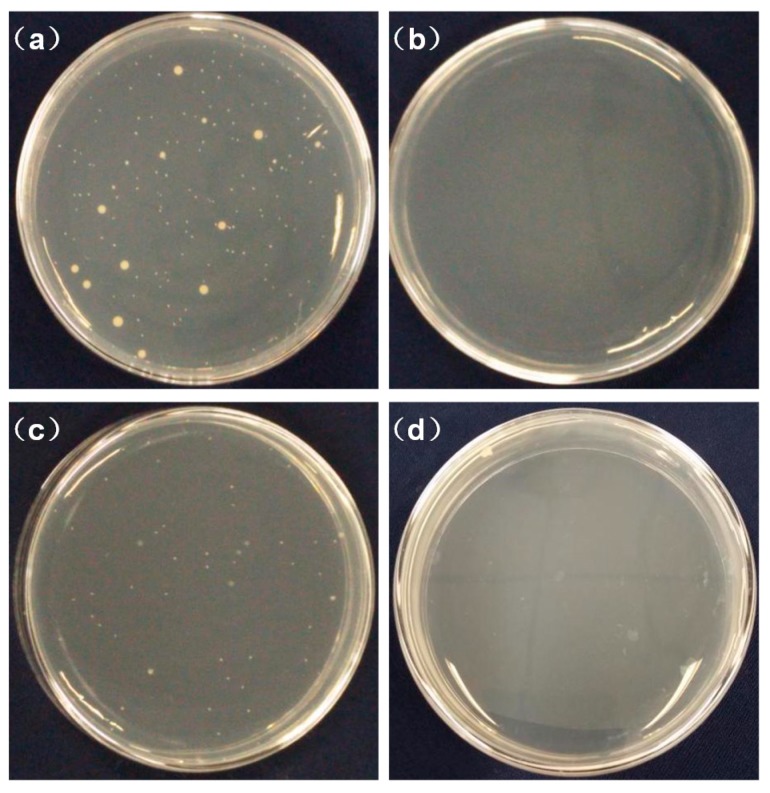
Antibacterial activity of the fibrous membranes against *S. aureus* (**a**,**b**), and *E. coli* (**c**,**d**). The samples are cotton (**a**,**c**), and fibrous membranes with 8 wt % PLA and 2.5 wt % CS (**b**,**d**).

*E. coli* is a gram-negative bacterium while *S. aureus* is a gram-positive bacterium. Both were used as model pathogenic bacteria to evaluate the effect of chitosan contents on antibacterial activity according to the national standard. As shown in Table 6, the antibacterial activity of nanofibre membranes was significantly enhanced with an increase in the chitosan content. PLA fibrous membranes without chitosan had a weak bacteriostatic effect, while the antibacterial activity of the PLA/chitosan membrane containing 1 wt % chitosan in the electrospinning solution increased remarkably. This is due to the presence of positively charged NH_2_ groups carried by chitosan, which are known to hinder biosynthesis and energy transport through the cell wall in order to kill bacteria [48]. It could be seen that when the mass ratio of chitosan to PLA was 2.5:8, the PLA/chitosan fibrous membrane exhibited an inhibition of 99.4% and 99.5% against *E. coli* and *S. aureus*, respectively, which is demonstrated in Figure 7. The results confirmed that the PLA/chitosan fibrous membranes exhibited excellent antibacterial capabilities.

### 3.6. Air Purification Performance

**Figure 8:**
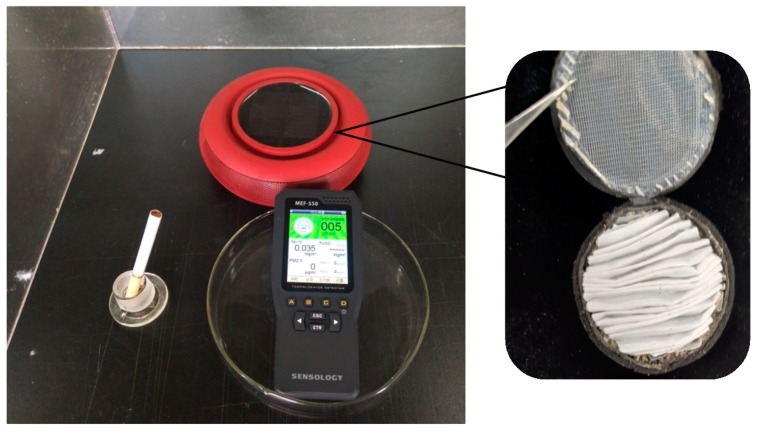
Apparatus of the air purification experiment, including car air purifier, air quality detector and cigarette. Enlarged figure on the right shows the filter in the car air purifier made of fibrous membranes.

**Figure 9:**
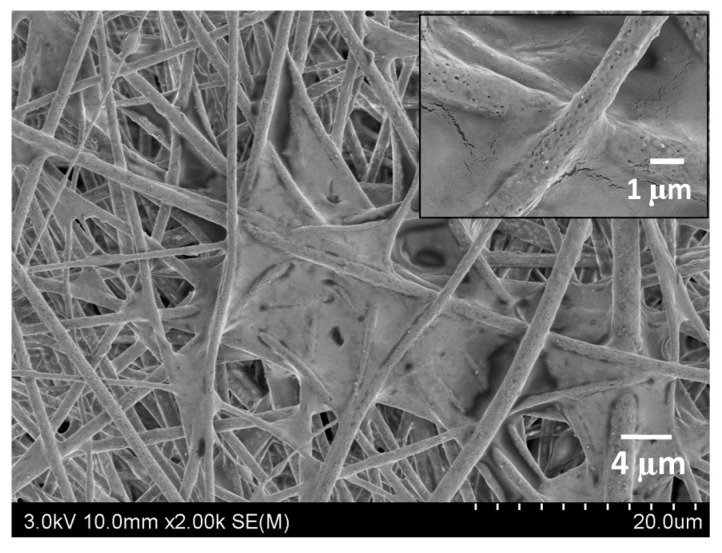
FE-SEM image of PLA/chitosan fibrous membranes after the air purification experiment.

Fibrous membranes will eventually be applied in the field of air purification. In order to study the air purification performance in a real polluted environment, a filter element for a car air purifier was prepared using the membrane (the apparatus is displayed in Figure 8). An artificially polluted environment was created by burning cigarettes in a confined space with a volume of 0.125 m^3^. The PM_2.5_ concentration tested by an air quality detector was initially over 999 μg/m^3^. The PM_2.5_ concentration decreased to 0 μg/m^3^ (PM_2.5_ removal efficiency of 100%) in 33 min when the PLA/chitosan fibrous membrane (the mass ratio of PLA to chitosan was 8:2.5) filter was used to purify the air. For comparison, the original filter included with the purifier required 44 min to achieve the same effect, which shows a purification efficiency that is 33% lower than that of the PLA/chitosan fibrous membrane. As shown in Figure 9, a large amount of particulate matter was deposited in the through-pores and on the surface of the fibres. Hence, the PLA/chitosan fibrous membrane could be used as an air filter medium with excellent purification performance.

## 4. Conclusions

In summary, an easy strategy for the preparation of porous composite fibrous air purification materials with excellent filtration and antibacterial performance has been demonstrated in this study. The porous PLA/chitosan fibrous membranes with different concentrations or different mass ratios of PLA to chitosan were prepared by dispersing the chitosan NPs in PLA/(DCM/DMAC) solutions. The solution concentration had a significant influence on the structure of the fibres. The fibres fabricated with low-concentration solutions consisted of bead-on-string structures. When the concentrations of PLA and chitosan were 8% and 2%, the fibres became even with no beads. A large number of nanopores was distributed on the surface of the fibres fabricated at over 30% humidity, while the width of the pores increased with the humidity. The different chitosan contents in the PLA solutions seem to have little impact on the morphology and through-pore structure, leading to a similar filtration efficiency and pressure drop. The membranes containing more chitosan certainly exhibited better antibacterial performance. The PLA/chitosan fibrous membranes (with a mass ratio of chitosan to PLA of 2.5:8) exhibited a high filtration efficiency of 98.99% and a relatively low pressure drop of 147.60 Pa. In addition, these membranes showed a high antibacterial activity of 99.4% and 99.5% against *E. coli* and *S. aureus*, respectively. The purification experiment further demonstrated that the fibrous membranes could form promising materials for air purification and consequently, other filtration applications.

## Figures and Tables

**Table 1:** Composition of electrospinning solutions.

Set	Sample	Chitosan/Solution wt %	PLA/Solution wt %	Mass Ratio of DCM/DMAC	Mass Ratio of Chitosan/PLA
A	A1	1	4	10:1	1:4
	A2	1.25	5	10:1	1:4
	A3	1.5	6	10:1	1:4
	A4	1.75	7	10:1	1:4
	A5	2	8	10:1	1:4
B	B1	0	8	10:1	0:8
	B2	1	8	10:1	1:8
	B3	1.5	8	10:1	1.5:8
	B4	2	8	10:1	2:8
	B5	2.5	8	10:1	2.5:8

**Table 2:** Characterisation of fibres prepared under various levels of humidity.

Humidity/%	Diameter/µm	Coverage of Pores/%	Width of Pores/nm
15	0.91 ± 0.14	/	/
30	1.15 ± 0.12	18.13	46.0 ± 12.8
45	1.34 ± 0.28	26.91	58.1 ± 14.8
60	1.50 ± 0.26	32.72	65.8 ± 15.9

**Table 3:** Characterization of fibres fabricated with different mass ratios.

Samples	Diameter/µm	Width of Pores/nm	Coverage of Pores/%	Mean Flow through-Pore Size/µm
0 wt % CS + 8 wt % PLA	1.21 ± 0.21	58.52 ± 18.97	23.22	4.50
1 wt % CS + 8 wt % PLA	1.34 ± 0.34	63.72 ± 18.02	23.85	7.25
1.5 wt % CS + 8 wt % PLA	1.31 ± 0.26	64.97 ± 16.32	24.46	7.53
2 wt % CS + 8 wt % PLA	1.35 ± 0.28	59.52 ± 20.57	23.81	7.92
2.5 wt % CS + 8 wt % PLA	1.35 ± 0.26	53.19 ± 20.39	23.57	7.72

**Table 4:** EDX analysis of the element contents in the materials.

Samples	C/wt %	N/wt %	O/wt %
100% PLA	70.13	0	29.87
100% chitosan	51.97	8.86	39.17
80% PLA/20% chitosan	60.45	2.75	36.50

**Table 5:** Filtration performance of fibrous membranes fabricated with different mass ratios.

Samples	Thickness/mm	Filtration Efficiency/%	Pressure Drop/Pa	Quality Factor
FY3107	1.152	89.18	71.67	0.0310
0 wt % CS + 8 wt % PLA	0.215	99.90	335.90	0.0207
1 wt % CS + 8 wt % PLA	0.196	98.10	167.05	0.0237
1.5 wt % CS + 8 wt % PLA	0.198	98.78	162.35	0.0271
2 wt % CS + 8 wt % PLA	0.206	98.26	147.00	0.0276
2.5 wt % CS + 8 wt % PLA	0.202	98.99	147.60	0.0312

**Table 6:** Antibacterial performance of fibrous membranes fabricated with different mass ratios.

Samples	Antibacterial Rate/%	
	*S. aureus*	*E. coli*
0 wt % CS + 8 wt % PLA	10.1	4.6
1 wt % CS + 8 wt % PLA	79.0	57.8
1.5 wt % CS + 8 wt % PLA	72.5	48.2
2 wt % CS + 8 wt % PLA	87.5	92.8
2.5 wt % CS + 8 wt % PLA	99.5	99.4
